# Exploring the Influential Factors on Readers' Continuance Intentions of E-Book APPs: Personalization, Usefulness, Playfulness, and Satisfaction

**DOI:** 10.3389/fpsyg.2021.640110

**Published:** 2021-02-19

**Authors:** Hehai Liu, Mingming Shao, Xiaohong Liu, Li Zhao

**Affiliations:** ^1^College of Education Science, Anhui Normal University, Wuhu, China; ^2^School of Education Science, Nanjing Normal University, Nanjing, China

**Keywords:** influential factors, undergraduates, continuance intention, e-book APPs, electronic book applications

## Abstract

With the rapid development of mobile devices, users can now read on the screen. Electronic reading (e-reading) has become a common reading style with the growth in online learning or electronic learning (e-learning). E-book applications (APPs) are widely developed and applied for reading on a screen. However, it is difficult for readers to change their reading habits or preference from paper-printed books to digital devices. The study of readers' continuance intention to use e-book APPs is the first step to improving e-reading. This study focuses on the influential factors on undergraduates' continuance intention of e-book APPs, which analyzed and summarized the literature related to the electronic book (e-book) applications (APPs) and undergraduates' continuous intention, combined with the characteristics of the e-book APPs, introduced relevant theories and variables, and established the factors that influence undergraduates' continuous intention of using e-book APPs. On this basis, the paper analyzed the relationship between various influencing factors and their influence on continuous intention. A model composed of five hypotheses was constructed to test the factors influencing undergraduates' continuous intention in e-book APPs. The results indicated that of all research variables, satisfaction is the most important factor that affects continuous intention; Perceived usefulness and perceived playfulness have an indirect effect on continuous intention through satisfaction; personalization has direct effects on perceived usefulness and perceived playfulness, so it also has an indirect effect on continuous intention. The findings of the study will be helpful for designers and developers of e-book APPs and provide e-book APP suggestions for readers as well.

## Introduction

The practice of reading is moving rapidly from print to screen (Miller and Warschauer, 2014). Recently, the emergence of social networks has enhanced the interaction between people and electronic devices (Su et al., 2019; Su and Chen, 2020). New media technologies are emerging one after the another. Reading texts is an unavoidable part of everyday life. Various electronic reading devices, such as Kindle, Sony Reader and electronic book (e-book) applications (APPs) such as iReader, are continuously emerging (Rainie et al., 2012). Compared with paper reading, the search ability and 24-h availability of electronic reading (e-reading) are popular among undergraduates (Jeong, 2012). More and more undergraduates choose this new way of reading (Coyle, 2008). Existing studies have shown that there is no difference in students' ability to understand what they read electronically or in print (Muter et al., 1982). Lim et al. (2020) also proposed that results from a middle school student reading comprehension test showed no statistical differences between the e-book reading group and the paper-based reading group. Although acceptance of e-reading is an important first step to success, actual success requires continued use. The concept of continuance intentions originates from the intention to act, which refers to an individual's subjective judgment on the possibility of a particular behavior in the future. It originates from the field of psychology. Researchers believe that consumers' will determine their behavior, thus introducing the concept of continuance intention (Bhattacherjee, 2001a). In summary, it is vital to investigate the factors on the undergraduates' willingness to continue using e-book APPs. It has great significance in satisfying demand and developing products for e-book APPs.

Early scholars used terms like “implement or execute” and “routine use” instead of the concept of “continuance intention.” Bhattacherjee (2001a) constructed the model of continuous use of information systems on the study of e-banking systems and proposed the term “continuance intention” for the first time, which he defined as the subjective tendency of users to continuously use e-banking. Thus, this study defined users' continuance intention of e-book APPs as: the likelihood or willingness of users of e-book APPs to continue using the APPs in the future.

In addition, a large number of scholars have conducted in-depth discussions on the users' continuance intentions in the field of information systems. Based on a model of technological acceptance, Venkatesh and Davis (2000) predicted user acceptance of computers by measuring their intentions. Perceived usefulness and perceived ease of use are considered to be key factors affecting the continuance intentions (Davis et al., 1989). Based on the study of online banking users' continuance intentions to use the information system, Bhattacherjee (2001a) proposed that the user's continuance intentions to use the information system depends on the satisfaction and perceived usefulness, and the degree of expected confirmation determines the satisfaction and perceived usefulness of users. Regardless, scholars have a lot of research on the continuance intentions in information systems, but there is little research on the continuance intentions in e-book APPs. In addition, existing studies have paid less attention to the role of personalization in the intention to continue using the product. Thus, this paper conducts a study on undergraduates' continuance intentions in e-book APPs. It also explores the correlation among personalization, perceived usefulness, perceived playfulness, satisfaction, and continuance intentions, and proposes suggestions for the improvement of e-book APPs.

## Literature Review

### E-books and E-reading Applications

With the increase of e-books, more and more people choose this new way of reading (Rainie et al., 2012). Since April 2011, there are 105 e-books for every 100 traditional paper books on Amazon. The authors detailed that print book sales would account for <25% of total sales (Miller and Bosman, 2011). With the text structured in a digital format (Muter et al., 1982), e-books are designed and built into an e-reading device or APP, such as Mobipocket, Microsoft Reader, or iReader. E-books have the same chapters and page numbers as paper books, however, readers can more easily navigate to any page or text and font type and size can also be modified according to the reader's preference. E-books have gained wider interest thanks to the introduction of portable e-readers and software-based readers, which provide a more authentic reading experience for users (Shiratuddin and Hassan, 2020). In recent years, Kindle, and Sony reader, set off a huge wave of e-reading. Today, e-reading devices have been replaced by e-reading APPs such iReader.

As for studies on e-book APPs, most scholars focus on the analysis of the relationship between traditional paper book reading and e-reading. A large number of scholars have proved that the effect of e-reading is no different or better than that of paper reading. In terms of reading comprehension, Muter et al. (1982) divided subjects into an e-reading group and a paper reading group and found that there was no difference in the understanding of the reading content between the two groups (Muter et al., 1982). Additionally, Kerr and Symons (2006) concluded that e-reading can recall more information freely through the comparison experiment of repetition between e-reading and paper reading (Kerr and Symons, 2006). Burghardt et al. (2009) investigated the differences between different reading carriers, such as paper and electronic devices, and concluded that the reading speed was related to individual factors, but not significantly to reading carriers (Burghardt et al., 2009). Recently, researchers have paid more attention to user experience, so the focus of research on e-reading has gradually shifted to practical research on e-reading and the design and development of related hardware and software technologies of e-reading equipment. A few studies have focused on undergraduates' continuance intentions of e-book APPs.

### Technology Acceptance Model

Davis (1989) first proposed the technology acceptance model. The TAM examines users' perceptions of usage, usefulness, and ease of use (Davis, 1989). According to this model, perceived usefulness and perceived ease of use can also directly or indirectly affect the user's behavior. Perceived usefulness refers to the actual utility of a new technology that users perceive to bring to their own lives, while perceived ease of use refers to the degree of difficulty users perceive when actually using this technology in operation.

Since then, more and more scholars have conducted research to explore people's acceptance of the product. Venkatesh et al. (2003) studied the theory of the technology acceptance model of development (TAM2), explaining perceived usefulness and the usage intentions in terms of social influence and cognitive instrumental processes (Venkatesh et al., 2003). He further designed a model which combined the technology acceptance model, the theory of planned behavior, the model of PC utilization, the innovation diffusion theory, and the social cognitive theory, called the unified theory of acceptance and use of technology (UTAUT). Dillon and Morris (2005) introduced the psychological factor, based on the user's acceptance of new information technology-theories (Dillon and Morris, 2005). Based on the TAM model, Fishbein and Ajzen (1977) increased four dimensions: social influence, facilitating conditions, playfulness, and trust (Fishbein and Ajzen, 1977). Ajwang et al. (2021) integrated three models of Innovation Diffuse (DOL), Technology Acceptance Model (TAM), and Technology Readiness Index (TRI) to enhance the understanding of factors that may affect the acceptance and use of smart waste management systems in smart cities. Wang et al. (2020) added personal innovation ability, environmental awareness, and perceived risk into the technology acceptance model to explore users' willingness to use ride-sharing services. Kong et al. (2020) also introduced the content of actual usage behavior into the technology acceptance model. Studies have shown that perceived usefulness, perceived ease of use, and trust factors of social media users will have a positive impact. Today, the technology acceptance model is widely used in research on mobile libraries and online learning.

Bhattacherjee (2000) follow-up studies show that user experience is more important after the initial use over time, and perceived usefulness has less of an impact. Thus, this study not only refers to the variables of perceived usefulness in the model, but also refers to the variables of perceived playfulness (Bhattacherjee, 2000).

### Theories About Perceived Playfulness

The theory of perceived playfulness was first proposed by Lieberman (1977), who believed that users would have feel entertained in the process of interacting with computers. Barnett (1991) defined perceived playfulness from the perspective of perceived playfulness characteristics and stated: (1) users' self-generated entertainment psychology is not affected by external factors; (2) an emotional state is induced by external factors (Barnett, 1991), respectively.

Moon and Kim (2001) first introduced the three variables of concentration, curiosity, and enjoyment to measure perceived playfulness while studying the extended technology acceptance model (Moon and Kim, 2001). Wherein, concentration refers to the user's interest during the process of using an information system, and other influences of the spirit of the time in the environment will be filtered. Curiosity means that the interest in the system is doubled in the interaction. The curiosity of the user is aroused and further exploration is promoted. Enjoyment refers to the user in the information system under the condition of interest, in the process of interaction with the information system rather than another external environment. Since it was first introduced, scholars continue to use perceived playfulness in relevant studies, and its influence has been tested in a large number of empirical studies. The research shows that perceived playfulness has a significant impact on both self-efficacy and subjective well-being (Román-Oyola et al., 2018; Demir, 2019).

### Literature Summary and Research Limitations

In general, at present, research on the technology acceptance model has matured, and many experts and scholars have expanded its application scope and variables when exploring the technology acceptance model. Most existing research however has applied it in the field of information systems, and not enough attention has been paid to new media. In addition, too much attention is paid to the influence of satisfaction on the continuous intention, and less to the personalized service of the product itself. Thus, based on the technology acceptance model, this current study took perceived usefulness and perceived playfulness as mediating variables to detect the correlation between personalization, satisfaction, and continuous intention to help improve the user's experience of e-reading.

### Research Model and Hypotheses

**Figure 1:**
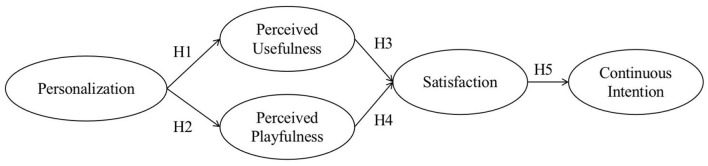
Model of undergraduates' continuance intention of using the e-book APP.

Based on related studies and theoretical discussions, the theory of expectation-confirmation (ECM) is taken as the theoretical basis in this study, and the study combined the technology acceptance model (TAM) and perceived playfulness theory. At the same time, according to characteristics of e-book APPs, five research variables were identified, including satisfaction (SAT), continuance intentions (CON), perceived usefulness (USE), perceived playfulness (PLA), and personalization (PER). A research model of undergraduates' continuance intention of using the e-book APP was constructed, as shown in Figure 1.

Dunne et al. (2010) used personalization as measurement variables to explore the reasons why young people use and participate in social networks. The study found that personalization can improve young people's satisfaction with social networks (Dunne et al., 2010). Liang et al. (2012) did a study on customer service provided by e-tailers. He found that personalization influences perceived usefulness through economic and emotional factors (Liang et al., 2012). Tan and Chou (2007) also explored factors on users' perceived playfulness toward mobile services of mobile service quality and its compatibility to mobile technology (Tan and Chou, 2007). They proposed that perceived playfulness was influenced by three most important service quality aspects, perceived usefulness, ease of use, and personalization. Al-Shboul et al. (2020) also proved that network personalization in product innovation has a positive impact on perceived usefulness and perceived ease of use. Therefore, this paper introduced related variables and designed H1 and H2.

H1: The personalized reading service of e-reading has a positive effect on undergraduates' perceived usefulness of the application.

H2: The personalized reading service of e-reading has a positive effect on undergraduates' perceived playfulness of the application.

The variable of perceived usefulness comes from the technical acceptance model proposed by Davis (1989). In the research on the continuous intention model of information systems, it is also proved that perceived usefulness is a key factor that directly affects user satisfaction and also has a direct impact on a user's willingness to continue using the product. Daud et al. (2018) also showed the impact of perceived usefulness on customer satisfaction in their use of IPTV. Thus, the variable of perceived usefulness was introduced to measure undergraduates' satisfaction with the e-book APP and their continuance intentions. H3 was proposed.

H3: undergraduate's perceived usefulness of using e-book APPs has a positive effect on their satisfaction with the APPs.

Lieberman (1977) first proposed the theory of perceived playfulness. Wang et al. (2009) introduced perceived playfulness into the research field of information systems. Users could improve their satisfaction because they feel happy in the process of using the information system, thus affecting their continuous intention. Tania and Marisol (2018) linked perceived entertainment and satisfaction through their research on art courses. Thus, in the study, H4 was proposed.

H4: undergraduates' perceived playfulness of e-book APPs has a positive effect on their satisfaction with the APPs.

Barnes and Böhringer (2011) explored users' continuance intention in microblogging services and found that perceived usefulness, satisfaction, and habit were the key factors (Barnes and Böhringer, 2011). Stone and Baker-Eveleth (2013) found that satisfaction and perceived usefulness influenced users' electronic textbook continuance usage intention (Stone and Baker-Eveleth, 2013). Satisfaction and continuance intention are also important variables in the expectation confirmation model (Amin et al., 2020). H5 was therefore proposed.

H5: undergraduates' satisfaction with e-book APPs has a positive effect on their continuance intentions of using the APPs.

## Methods

The research data collection used a questionnaire. The questionnaire consisted of four parts. The first part was the explanatory information of the questionnaire. The second part was a survey on the participants' basic information (three items). The third part was the participants' experience with e-book APPs (three items). The fourth part was the investigation on the factors influencing undergraduates' continuous intention of e-book APPs, which was the main part of the questionnaire and was designed in the form of a 5-level Likert scale. The options of each question included five levels, indicating the respondents' recognition of the question, so as to explore the factors affecting undergraduates' continuous intention in e-book APPs. The original questionnaire has 5 latent variables, perceived usefulness (4 items), perceived playfulness (5 items), personalization (5 items), satisfaction (4 items) and continuance intentions (5 items), a total of 23 items.

The survey was distributed in two stages. In the first stage, based on the existing research, to test the reliability and validity of the questionnaire, a survey among students from two classes in the fourth grade of an Education Technology major in a Chinese university was conducted. SPSS 24.0 statistical software showed that the reliability and validity of the questionnaire were good. Combined with the test results, two questions were deleted. The questionnaire was modified as 21 items.

In the second stage, the survey was distributed in a large scale and was divided into online and offline parts. The online part was distributed with the tool called *Questionnaire Stars*, and the offline part was distributed and recovered in public places such as libraries and study rooms of three universities in Nanjing, China. In the explanatory part of the questionnaire, the subjects were given a questionnaire “if you have ever used or are currently using e-book APPs, please continue reading and finish the questionnaire, but if you have never used any e-book APPs, please stop answering.” This declaration filters out undergraduates who are not currently using e-book APPs. In this survey, 133 online questionnaires and 286 paper questionnaires were recovered, totaling 429. After sorting out the online questionnaires and paper questionnaires, 421 valid questionnaires were obtained, and the effective recovery rate reached 98%.

## Data Analysis and Results

In this study, SPSS 24.0 was used for descriptive statistics and correlation analysis of population information. Male undergraduates accounted for 33.7% of the participants and female undergraduates accounted for 66.3% of the participants. 44.3% were majoring in liberal arts, 53.6% were majoring in science, and only 2.1% were studying arts. The participants included 13.8% freshmen, 24.4% sophomores, 49.2% juniors, and 12.6% seniors.

The questionnaire listed the top eight popular e-book APPs as options, such as QQ reading, book reading novels, palm reading iReader, baidu reading, Migu reading, WeChat reading, netease cloud reading, and douban reading. The “other” option was also designed for users. The data showed that some users used other APPs as well. Most of them used Kindle. A small number of the group used starting point reading, ibooks, super star readers, and other APPs.

In addition, the duration and frequency of undergraduates' use of e-book APPs were also investigated and counted. According to the survey, there were 115 users and 112 users who used e-book APPs for 21–40 min and more than 1 h, respectively, accounting for 27.3 and 26.6% of the sample data, while the users who used for 41–60 min were the least, with only 23 users, accounting for 5.5% of the sample. The reading frequency of users showed that the number of users reading once a week or less and every 2 or 3 days was large—a total of 290 people—accounting for 56.5% of the total sample.

### Reliability and Validity of the Instrument

The data analysis of this study is divided into three stages. The first stage is first-order CFA to determine the structure and internal reliability of the tool. The second stage is confirmatory factor analysis (CFA) to further confirm the convergence and discriminability of the construction. In the third stage, the structural equation modeling (SEM) is used to evaluate the hypothetical structural model. AMOS (24.0 edition) was used in this study to conduct CFA and path analysis on the structural model of questionnaire data.

In this study, a first order confirmatory factor analysis was used to remove the problems with low reliability and dimensional confusion, so as to facilitate the fitting degree of the model in the later period, if the standardized load of the measurement is <0.5, the measurement should be deleted (Hair et al., 2014). Absolute fitting index and relative fitting index are used to evaluate the fitting degree of the model. After removing some of the questions, in this model, the chi-square is 116/501, the degree of freedom is 109, and the chi-square/df is 1.069. The value of the root mean square error of approximation (RMSEA) of this study was 0.013 (RMSEA <0.08) (Anderson and Gerbing, 1988). The value of goodness-of-fit index (GFI) and the adjust fitness index (AGFI) was 0.970 and 0.958 (0.9 < GFI <0.1, 0.9 < AGFI <0.1) (Foster et al., 2006). The value of normed fit index (NFI), comparative fit index (CFI), and the relative fitness index (RFI) was 0.981, 0.999, and 0.976 (0.9 < NFI <0.1, 0.9 < CFI <0.1, 0.9 < RFI <0.1) (Hair et al., 1995).

From the perspective of model indexes, the chi-square/df, RMSEA, GFI, AGFI, NFI, CFI, and IFI are all within the acceptable range. Hence, 17 remaining items were kept for further analysis, including perceived usefulness (4 items), perceived playfulness (3 items), personalization (3 items), satisfaction (4 items) and continuous use intention (3 items) (see Appendix 1).

### Dimension Reliability and Validity Analysis

To ensure the reliability and validity of the scale, we used Cronbach's alpha to test its reliability, CR value to test its validity, and the AVE value to test its convergence. According to the Cronbach's alpha reliability standard, when Cronbach's alpha was bigger than 0.7, the questionnaire was credible (Bagozzi and Yi, 1988). In this model, the value of Cronbach's alpha this are all more than 0.8. For a more accurate measurement, the composite reliability (CR) is the reliability of all measurement items, and it is generally suggested that a good value should be >0.7 (Hair et al., 2014). In this study, CR values ranged from 0.862 to 0.947. To ensure the validity of the measurement model and at the same time, when the AVE (Average Variance Extracted. AVE) value exceeds 0.5, it indicates that the dimension has the effect of convergence (Fornell and Larcker, 1981), which means that the load of all standardized factors in the dimension is squared, aggregated, and finally averaged. If the calculated value meets this standard, the convergence validity of each dimension is significant enough. Thus, as Table 1 shows, the model of this study was constructed reasonably, and the questionnaire had a high validity.

**Table 1:** Results of confirmatory factor analysis.

**Latent variable**	**Measure item**	**Average**	**Standard deviation**	**Standardized factor loading**	**CR**	**AVE**	**Cronbach's alpha**
Critical value				>0.5	>0.7	>0.5	>0.7
Perceived usefulness (USE)	USE1	3.42	0.866	0.868	0.9231	0.7502	0.923
	USE2	3.58	0.817	0.880			
	USE3	3.54	0.879	0.823			
	USE4	3.65	0.810	0.892			
Perceived playfulness (PLA)	PLA1	3.15	1.082	0.789	0.8624	0.6765	0.861
	PLA2	3.10	1.001	0.860			
	PLA3	3.24	0.982	0.817			
Personalization (PER)	PER1	3.01	0.984	0.818	0.9136	0.7794	0.912
	PER2	3.28	0.934	0.904			
	PER3	3.29	0.933	0.923			
Satisfaction (SAT)	SAT1	3.43	1.158	0.917	0.947	0.8171	0.949
	SAT2	3.41	1.211	0.921			
	SAT3	3.41	1.201	0.922			
	SAT4	3.43	1.195	0.854			
Continuous use intention (CON)	CON1	3.54	0.969	0.889	0.880	0.7103	0.882
	CON2	3.23	0.963	0.853			
	CON3	3.20	0.932	0.783			

*CR, composite reliability; AVE, average variance extracted*.

After checking the convergence degree of the scale, we should check the discriminant validity of the model to ensure that the questions in each dimension are independent of those in other dimensions. In terms of construct discriminant validity analysis (see Table 2). Normally, the square root of AVE of each dimension must be obtained first, which must be greater than the absolute value of Pearson correlation coefficient between the two dimensions before each dimension can be expressed as discriminant validity (Schumacker and Lomax, 2016). In this study, the square root of the AVE value of all variables is greater than the absolute value of correlation coefficient between variables, which proves that the measurement model has good discriminative validity (Schumacker and Lomax, 2016).

**Table 2:** Correlation coefficient matrix and square roots of AVE.

**Construct**	**USE**	**PLA**	**PER**	**SAT**	**CON**
USE	0.866				
PLA	0.307^**^	0.822			
PER	0.404^**^	0.256^**^	0.883		
SAT	0.606^**^	0.421^**^	0.473^**^	0.904	
CON	0.598^**^	0.326^**^	0.422^**^	0.743^**^	0.843

** *p <0.01*.

### Hypotheses Testing

In this study, absolute fitting index and relative fitting index are used to evaluate the fitting degree of the model. The goodness-of-fit index (GFI) should be more than the suggested value of 0.9 and <1.0 (Foster et al., 2006). The value of GFI was 0.952. The normed fit index (NFI) and comparative fit index (CFI) should all be >0.9 (Hair et al., 1995). The value of NFI was 0.969 and CFI was 0.987. The root mean square error of approximate (RMSEA) should be <0.08 (Dillon et al., 1988), and the value of this study model is 0.039. From the perspective of model indexes, as shown in Table 3, the chi-square value and degree of freedom ratio, the approximate error mean square value, the goodness of fit index (GFI), the model comparative fitness index (CFI), the non-normal fit index (NFI), and the incremental fitness index (IFI) were all within the acceptable range. Therefore, the model used for this study fits well.

**Table 3:** Model fitting analysis results.

**Type**	**Fitting index**	**Evaluation standard**	**The fitting results of this model**	**Results**
Absolute fit index	Chi-square/df	<3	1.650	Supported
	RMSEA	<0.08	0.039	Supported
	Goodness-of-fit index (GFI)	>0.9	0.952	Supported
	Adjust goodness fitness index (AGFI)	>0.9	0.936	Supported
Relative fit index	Normed fitness index (NFI)	>0.9	0.969	Supported
	Non-normalized fitness index (NNTI/TFI)	>0.9	0.985	Supported
	Comparative fitness index (CFI)	>0.9	0.987	Supported
	Incremental fitness index (IFI)	>0.9	0.988	Supported
	Relative fitness index (RFI)	>0.9	0.963	Supported
Parsimonious fit index	Parsimonious normed fitness index (PNFI)	>0.5	0.812	Supported
	Parsimonious goodness fitness index (PGFI)	>0.5	0.710	Supported

Through the path analysis of the relationship between variables, this study tested the hypothesis of the research model. The analysis results of path coefficient are shown in Table 4. It can be seen that the significance of the five hypotheses proposed in this study has been verified. The *p*-value was <0.01.

**Table 4:** Path coefficient analysis results.

**Hypothesis**	**Causal factors**	**Estimate**	**S.E**.	**C.R**.	***p***
H1	USE← PER	0.375	0.042	5.884	^***^
H2	PLA← PER	0.294	0.050	8.972	^***^
H3	SAT← USE	0.832	0.063	13.236	^***^
H4	SAT← PLA	0.355	0.054	6.615	^***^
H5	CON← SAT	0.686	0.039	17.703	^***^

*** *p <0.001*.

**Figure 2:**
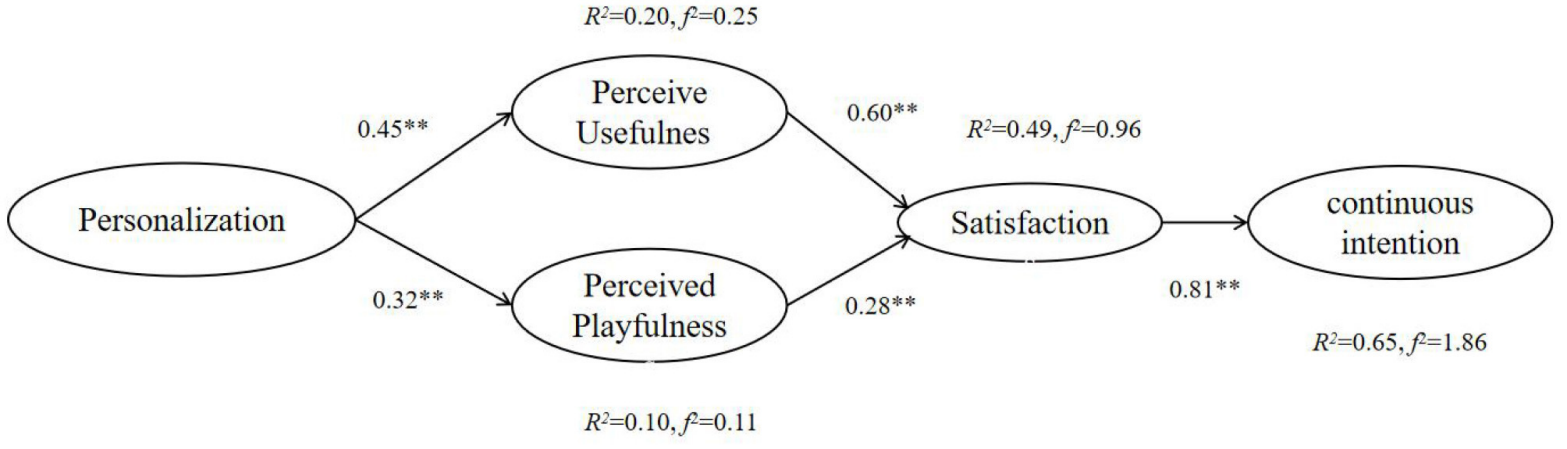
The final structural model with standardized coefficients. **p < 0.01.

In order to improve the fitting degree of the model, the following adjustments were made, as shown in Figure 2.

The determination coefficient *R*^2^ quantifies the variance ratio interpreted by the statistical model and is an important summary statistic of biological benefits (Nakagawa et al., 2017). *R*^2^ represents the degree of interpretation between paths. We consider that *R*^2^ values 0.3–0.6 is medium, and <0.3 is low (Sanchez and Golding, 2013). In addition, effect size of the model (*f* ^2^) was proposed by Cohen (1988). This allows researchers to move from a simple recognition of statistical significance to a more general quantifiable description of the size of the effect (Fritz et al., 2012). *f* ^2^ >0.8 can be considered large. When it is between 0.2 and 0.8, it can be considered medium, and when it is <0.2, it can be considered small. In this study, the explanatory power of PER on USE is 20% (*R*^2^ = 0.20, *f* ^2^ = 0.25), and PLA is 10% (*R*^2^ = 0.10, *f* ^2^ = 0.11). The explanatory variance of USE and PLA on SAT is 49.0% (*R*^2^ = 0.49, *f* ^2^ = 0.96). The explanatory variance of SAT on CON is 65.0% (*R*^2^ = 0.65, *f* ^2^ = 1.86). Hence, the six variables in this study have good predictive power (Hair et al., 2014). To improve the fitting degree of the model, the following adjustments were made, as shown in Figure 2.

In this study, the direct and indirect influences of each factor on the intention of continuous use were calculated according to the path coefficient, and their influence coefficients were combined to obtain the influence, so as to observe the effect of each research variable on the intention of continuous use of undergraduates' e-book APPs.

Table 5 shows the degree of each variable of influencing factors of undergraduates' intention to continue using e-book APPs investigated in this research model, as personalization (0.289), perceived usefulness (0.485), perceived playfulness (0.230), and satisfaction (0.808).

**Table 5:** Analysis on the effect of each variable.

**Variable**	**Directly influence**	**Indirectly influence**	**Totally influence**	**comprehensive sequencing**
Personalization (PER)		0.289	0.289	3
Perceived usefulness (USE)		0.485	0.485	2
Perceived playfulness (PLA)		0.230	0.230	4
Satisfaction (SAT)	0.808		0.808	1

### Moderated Mediating Effect Analysis

First, Model 4 of the SPSS macro compiled by Hayes (2012) (Model 4 is a simple mediation model) was used to test the mediating effect of satisfaction in the relationship between perceived usefulness, perceived playfulness, and continuous intention. As Table 6 shows, the results showed that perceived usefulness had a significant predictive effect on continuous intention (β =0.0335, *t* = 5.8913, *p* < 0.01), and the direct predictive effect was still significant when the mediator variable was included (β = 0.0229, *t* =15.2495, *p* < 0.01). At the same time, the mediating effect of satisfaction between perceived entertainment and intention to use continuously is not significant (β = 0.0341, *t* = 0.6582, *p* >0.01).

**Table 6:** The mediation model testing of satisfaction.

**Outcome variable**	**Predictor variable**	**Fitting index**			**Coefficient significance**		
		***R***	***R*^**2**^**	***F*_**(df)**_**	**Coeff**	**se**	***t***
SAT		0.6058	0.3669	242.8736			
	USE				0.8849	0.0568	15.5844^**^
CON		0.7662	0.5870	27.0522			
	USE				0.1973	0.0335	5.8913^**^
	SAT				0.3496	0.0229	15.2495^**^
SAT		0.4188	0.1696	85.5582			
	PLA				0.6739	0.0729	9.2498^**^
CON		0.7438	0.5532	258.7389			
	PLA				0.0224	0.0341	0.6582
	SAT				0.4258	0.0208	20.4502^**^

** *p <0.001*.

In addition, as Table 7 shows, the upper and lower limits of the Bootstrap 95% confidence interval of the direct effect of perceived usefulness on continuous intention and the mediating effect of satisfaction do not contain 0, indicating that perceived usefulness can not only directly predict continuous intention, but can also predict continuous intention through the mediating effect of satisfaction. The direct effect (0.197) and the mediating effect (0.309) accounted for 38.86 and 60.95% of the total effect (0.507), respectively.

**Table 7:** Total effect, direct effect, and mediating effect.

	**Effect**	**Boot SE**	**BootLLCI**	**BootULCI**	***t***
Total effect	0.5067	0.0332	0.4414	0.5720	15.201^**^
Direct effect	0.1973	0.0335	0.1315	0.2631	5.8913^**^
Mediating effect of SAT	0.3094	0.0273	0.2571	0.3664	

** *p <0.001*.

## Discussion and Conclusion

Based on the continuance intentions model, this study introduced relevant theoretical variables and constructed the users' continuance intention model of undergraduates' e-book APPs. According to the model hypotheses, the questionnaire was designed, distributed, and recovered. After statistical analysis and obtaining effective data, the rationality of the research model was verified. Furthermore, the influence of various research variables on undergraduates' continuance intentions for using e-book APPs was analyzed.

This study proposed five factors; personalization, perceived usefulness, perceived playfulness, and satisfaction will have a direct or indirect influence on undergraduates' continuance intentions in e-book APPs. The empirical hypothesis test confirmed that the five factors had a significant impact.

The satisfaction has a positive direct impact on users' continuance intentions, and its direct impact load was 0.860, the most important of all the variables in this study. H5 was proved. Chiu et al. (2007) and Amin et al. (2020) also proved the point in their studies. Perceived usefulness and perceived playfulness could have a positive indirect effect on the satisfaction of undergraduates' continuance intentions of using e-book APPs by 0.485 and 0.230. The total effect of 0.485 and 0.230 ranked the second and fourth among all variables in this study. So H3 and H4 were proved. Which were the same as the studies by Daud et al. (2018) and Tania and Marisol (2018). However, the conclusion was different from Almaghrabi's study (Almaghrabi and Dennis, 2012).

In addition, the variable of personalization also had a direct effect on perceived usefulness and perceived playfulness reaching 0.447 and 0.316. At the same time, personalization had an indirect effect on undergraduates' continuance intentions reaching 0.289. The total effect of 0.289 ranked the third among all variables in this study. So H1 and H2 were proved. Liang et al. (2012) and Tan and Chou (2007) reached the same conclusion (Lee and Kim, 2005; Moller, 2015). So as the study of Al-Shboul et al. (2020).

This study shows that the most important factor influencing undergraduates' willingness to continue using e-book APPs is satisfaction, followed by perceived usefulness, personalization, and perceived playfulness. The research results show that e-book APPs should satisfy users' perceived usefulness first. Secondly, personalization is particularly important, which is also a common problem of e-book APPs in the market. It is necessary to understand the personalized needs of users from the sense of the user experience, such as the different functional requirements and appearance requirements of users' different professions, and then it is necessary to realize these in terms of technology and involvement. In addition, the perception and entertainment of products should not be ignored. Attention should also be paid to the smooth use and beautiful interface for the e-book APPs.

In addition, the majority of undergraduates' continuance intentions of using e-book APPs depended on the fact that e-book APPs could make them feel satisfied with the process and experience, then the user was likely to choose to continue to use the application. The e-book APPs could make the user feel expected and confirmed. If users' experience of using e-book APPs met the expected value or even exceeds it, they would have the willingness to continue using the APP. Moreover, if the e-book APP could increase the user's reading volume, improve the reading efficiency, and reasonably allocate the time, the user would also have the intention to continue to use the e-book APP.

The opinions and feelings of people and comments from the outside media would affect users' continuance intentions of using a product. How to improve users' subjective standards is therefore also a problem that should be considered in e-book APPs.

## Limitation and Future Study

The samples collected in this study have a limited coverage and are concentrated in different regions. Therefore, they cannot fully represent all undergraduates' continuance intention of using e-book APPs. Future research can hopefully appropriately increase the number of samples, expand the sample range, and carry out more accurate and comprehensive research.

Users' continuous intention of using a product is a dynamic and continuous process. Deep learning and mining of data is especially necessary (Su et al., 2020, 2021; Su and Wu, 2021). There will hopefully be an opportunity to conduct a more long-term dynamic study in the future, which will continue to optimize and improve the model based on more data.

## Data Availability Statement

The raw data supporting the conclusions of this article will be made available by the authors, without undue reservation.

## Ethics Statement

Ethical review and approval was not required for the study on human participants in accordance with the local legislation and institutional requirements. Written informed consent for participation was not required for this study in accordance with the national legislation and the institutional requirements.

## Author Contributions

All authors contributed equally to the conception of the idea, implementing and analyzing the experimental results, writing the manuscript, and read and approved the final manuscript.

## Conflict of Interest

The authors declare that the research was conducted in the absence of any commercial or financial relationships that could be construed as a potential conflict of interest.

## Appendix 1

**Table A1:** Questionnaire of variables.

**Construct**	**Items**	**References**
Perceived usefulness (USE)	USE1: Using e-book APPs has increased the amount of my reading and improved my reading efficiency. USE2: Using e-book APPs broadened my reading range. USE3: Using e-book APPs allows me to spend my time more efficiently and read more freely. USE4: In general, using e-book APPs is useful for me.	Davis et al., 1989
Perceived playfulness (PLA)	ENT1: Reading with an e-book APP makes me happy. ENT2: When I use an e-book APP for reading, I feel time flies. ENT3: The use of e-book APPs for reading has increased my interest in reading, which is an enjoyable thing for me.	Barnett, 1991; Moller, 2015
Personalization (PER)	IND1: The personalized recommendation content and frequency of e-book APPs are what I need. IND2: The e-book APPs personalized Settings meet my personalized needs. IND3: In a word, the e-book APPs meet my personal reading needs.	Lee and Kim, 2005
Satisfaction (SAT)	SAT1: I enjoy and satisfying with using e-book APPs. SAT2: I am satisfied with the process and experience of using e-book APPs. SAT3: For me, using e-book APPs is a smart choice. SAT4: Overall, I'm happy with the e-book APPs.	Bhattacherjee, 2000; Bhattacherjee and Premkumar, 2004; Hong et al., 2006
Continuous use intention (CON)	CON1: I will continue to use the e-book APPs. CON2: I intend to continue to use e-book APPs rather than related alternative businesses or services. CON3: I will recommend e-book APPs to my friends and family	Bhattacherjee, 2001a,b
